# Protein Arginine Methyltransferase 5 as a Therapeutic Target for *KRAS* Mutated Colorectal Cancer

**DOI:** 10.3390/cancers12082091

**Published:** 2020-07-28

**Authors:** David Shifteh, Tzuriel Sapir, Moshe Pahmer, Adam Haimowitz, Sanjay Goel, Radhashree Maitra

**Affiliations:** 1 Department of Biology, Yeshiva College, Yeshiva University, 500 W 185th St, New York, NY 10033, USA; dshifteh@mail.yu.edu (D.S.); tsapir@mail.yu.edu (T.S.); mpahmer@mail.yu.edu (M.P.); adam.haimowitz@einsteinmed.org (A.H.); 2 Department of Oncology, Albert Einstein College of Medicine, Montefiore Medical Center, 1300 Morris Park Avenue, Bronx, NY 10461, USA; sgoel@montefiore.org

**Keywords:** CRC, PRMT5, *KRAS*, EPZ015666, Arginine methylation

## Abstract

Nearly 45% of colorectal cancer (CRC) patients harbor a mutation in their *KRAS* gene for which, despite many years of research, there are still no targeted therapies available. Protein Arginine Methyltransferase 5 (PRMT5) is a transcription regulator for multiple cellular processes that is currently being tested as a potential target in several cancer types. PRMT5 has been previously shown to be overexpressed in approximately 75% of CRC patient tumor samples, as well as negatively correlated with CRC patient survival. Here, we provide evidence that PRMT5 can act as a surrogate target for mutated *KRAS* in CRC. Our findings show that PRMT5 expression is upregulated, as well as positively correlated with *KRAS* expression, in CRC patient datasets. Moreover, our results reveal that PRMT5 is further overexpressed in *KRAS* mutant CRC cells when compared to *KRAS* wild type (WT) CRC cells at both the transcriptional and translational levels. Additionally, our data demonstrate that this further overexpression of PRMT5 in the *KRAS* mutant CRC cells affects an even greater degree of growth inhibition, apoptosis, and cell cycle arrest, following treatment with PRMT5 inhibitor, when compared to the *KRAS* WT CRC cells. Our research therefore suggests for the first time that PRMT5 and *KRAS* may crosstalk, and thus, PRMT5 can potentially be used as a surrogate target for mutated *KRAS* in CRC.

## 1. Introduction

Colorectal cancer (CRC) is the second largest cause of cancer death in the USA [1]. It is estimated that, in 2020, there will be 147,950 new diagnoses and 53,200 new deaths of colorectal cancer in the USA alone [1]. Most cases of CRC take over ten years to fully develop and advance through the adenoma-carcinoma sequence [2,3]. Inactivating mutations of the *APC* tumor suppressor gene are an early step in the development of CRC and occur in over 70% of colorectal adenomas [2,4]. Additional activating mutations of the *KRAS* oncogene and inactivating mutations of the *TP53* tumor suppressor gene further promote the adenoma-carcinoma sequence [2,4].

Epidermal growth factor receptor (EGFR) is a transmembrane receptor tyrosine kinase (RTK) that is responsible for sending a downstream signal to initiate growth-promoting events [5]. When EGFR is overexpressed, excessive cellular proliferation results [6]. Cetuximab, a monoclonal antibody that binds to the extracellular domain of EGFR and prevents ligands from binding, was therefore developed as a treatment for CRC [7]. Cetuximab, however, was later determined to be detrimental for CRC patients with a mutated *KRAS* gene [8,9]. 

*KRAS* is a membrane-bound GTPase that plays an early and lead role in many signal transduction pathways [10]. Activating mutations in *KRAS* have been shown to result in increased cellular proliferation as well as suppression of apoptosis [11,12]. As *KRAS* has been determined to be mutated in around 45% of CRC patients, much research has been pursued to develop an inhibitor for mutant *KRAS* [13]. However, despite many years of research, there are still no therapies available that target mutant *KRAS* in CRC [14,15]. Other treatments that indirectly target mutant *KRAS* are thus urgently sought after in the clinical setting.

Arginine methylation is an important post-translational modification that functions as an epigenetic regulator for multiple cellular processes including gene expression, protein modification, signal transduction, and cell cycle progression [16,17]. Arginine methylation is catalyzed by a group of enzymes known as the protein arginine methyltransferases (PRMTs) [18]. PRMTs use a cosubstrate called *S*-Adenosyl methionine (AdoMet) as a methyl donor to catalyze the transfer of methyl groups to the arginine residues of histone and non-histone proteins [18,19]. Increasing evidence indicates that PRMTs play a critical role in cancer progression and maintenance [20,21]. 

PRMT5 is a protein arginine methyltransferase that catalyzes the dimethylation of histones H3 (H3R8me2s) and H4 (H4R3me2s), resulting in chromatin restructuring, which in turn promotes transcriptional repression [18,20]. Moreover, PRMT5 has been found to additionally regulate gene expression through the methylation of key transcription factors such as p53 and NF-κB [18,22,23]. Previous studies have shown that PRMT5 is overexpressed in several types of cancers, and that PRMT5 inhibition and knockdown resulted in a substantial decrease in cellular proliferation, migration, and colony-forming abilities, as well as increases in apoptosis and cell cycle arrest, in both in vitro and in vivo studies [24,25,26]. In light of these findings, PRMT5 is currently being tested as a potential therapeutic target in several cancer types [24,26].

In CRC, PRMT5 has been found to be overexpressed in approximately 75% of patient tumor samples, as well as being negatively correlated with patient survival [27]. PRMT5 inhibition and knockdown have also been shown to restore key regulatory pathways that are involved in cell growth, survival, migration, and tumor suppressor activity [27]. 

In this study, we aim to determine whether PRMT5 can act as a surrogate target for mutated *KRAS* in CRC. We first show that PRMT5 expression is upregulated, as well as positively correlated with *KRAS* expression, in CRC patient datasets. We next show that PRMT5 is further overexpressed in *KRAS* mutant CRC cells when compared to *KRAS* WT CRC cells at both the mRNA and protein levels. We then show that this further overexpression of PRMT5 in the *KRAS* mutant CRC cells results in more substantial growth inhibition, apoptosis, and cell cycle arrest following PRMT5 inhibitor treatment when compared to the *KRAS* WT CRC cells. We therefore propose for the first time that PRMT5 and *KRAS* may crosstalk, and thus, PRMT5 may be able to act as a surrogate target for mutated *KRAS* in CRC.

## 2. Materials and Methods

### 2.1. Cell Lines

Six CRC cell lines—HCT116, SW620, Caco-2, HT-29, HKe3, and LIM2405—were used in this study and were tested to be negative for mycoplasma contamination by Venor™ GeM Mycoplasma Detection Kit (Sigma-Aldrich™, St. Louis, MO, USA, Catalog #: MP0025), as well as authenticated by clustering analysis of genome-wide mRNA expression microarray data. HCT116, SW620, Caco-2, and HT-29, as well as Normal colon epithelial cell line CCD 841 CoN were purchased from the American Type Culture Collection (ATCC^®^, Manassas, VA, USA). LIM2405 and HKe3 cell lines were obtained from Dr. Robert Whitehead (Ludwig Institute for Cancer Research) and Dr. Takehiko Sasazuki (Medical Institute of Bioregulation, Kyushu University), respectively. HCT116 and SW620 are *KRAS* mutant cell lines, whereas Caco-2, HT-29, HKe3, and LIM2405, are *KRAS* WT cell lines. The HCT116 cell line harbors a G13D *KRAS* mutation, while the SW620 cell line harbors a G12V *KRAS* mutation [28]. HCT116 and HKe3 are isogenic cell lines: Hke3 cells were produced from HCT116 cells by correcting for the mutant *KRAS* by homologous recombination, as has been previously described by Shirasawa S [29].

### 2.2. Cell Culture

HCT116, SW620, HT-29, HKe3, LIM2405, and CCD 841 CoN were all cultured in Minimum Essential Medium (MEM) (Corning™, Corning, NY, USA, Catalog #: 10010CV) with 10% Fetal Bovine Serum (FBS) (GemCell™, West Sacramento, CA, USA, Catalog #: 100-500), 1% Non-Essential Amino Acids (NEAA) (Gibco™, Grand Island, NY, USA, Catalog #: 11140050), and 2% HEPES buffer (Lonza™, Basel, CH, Catalog #: BW17737E) (final concentration of 20 mM). Caco-2 was cultured in MEM with 20% FBS, 1% NEAA, and 2% HEPES buffer. While many standard cell culture protocols explicitly call for the addition of antibiotics/antimycotics to the cell culture media, we followed the recommendations of the ATCC^®^ and Ann H. Ryu to avoid the use of antibiotics/antimycotics in cell culture media [30,31]. The cells were maintained in an atmosphere of 5% CO_2_ at 37 °C and passaged according to ATCC^®^’s recommended protocol.

### 2.3. Gene Expression Profiling Interactive Analysis (GEPIA)

The GEPIA online website was used to analyze the RNA-Seq data of CRC patients from The Cancer Genome Atlas (TCGA) database [32]. We first compared the PRMT5 expression of CRC patient tumor samples with normal colon and rectum tissue. This was done by logging onto the GEPIA website and selecting the Single Gene Analysis tab and inserting PRMT5. The Expression DIY Profile tab was then selected to arrive at Gene Expression Profile and the following options were then chosen: Gene = PRMT5, Differential Methods = ANOVA, |Log_2_FC| Cutoff = 1, q-value Cutoff = 0.01, Log Scale = No, Matched Normal data = Match TCGA normal and GTEx data, Dataset = COAD & READ, and Plot Width = 12. We then assessed the correlation between PRMT5 and *KRAS* gene expression. This was done by logging onto the GEPIA website and selecting the Multiple Gene Analysis tab, selecting Correlation Analysis, and then selecting the following options: Gene A = PRMT5, Gene B = *KRAS*, Normalized by gene = TUBA1A, Correlation Coefficient = Spearman, and Used Expression Datasets = COAD Tumor & READ Tumor.

### 2.4. RNA Extraction 

HCT116, SW620, Caco-2, HT-29, and CCD 841 CoN cells were cultured until 70% confluency. The cells were then trypsinized (Corning™, Catalog #: 25-053-CI) and subsequently spun down into cell pellets. RNA was then extracted from the cell pellets using the Invitrogen™ PureLink™ RNA Mini Kit (Invitrogen™, Carlsbad, CA, USA, Catalog #: 12183018A) as per the manufacturer’s protocol. The cell lysates were homogenized by being passed five times through 21-gauge tuberculin syringes (BD™, Franklin Lakes, NJ, USA, Catalog #: 309624) as per the manufacturer’s (Invitrogen™) recommendation. On-column DNase I digestion was also performed using the Invitrogen™ PureLink™ DNase Set (Invitrogen™, Catalog #: 12185010) as per the manufacturer’s protocol. The purified RNA was then placed into single-use aliquots and stored at −80 °C.

### 2.5. RNA Quantification

The concentration of the extracted RNA was quantified using an Invitrogen™ Qubit™ RNA BR Assay Kit (Invitrogen™, Catalog #: Q10211) as per the manufacturer’s protocol.

### 2.6. cDNA Synthesis

A total of 1 μg of the extracted and quantified RNA was synthesized into cDNA using the Maxima™ H Minus cDNA Synthesis Master Mix (Thermo Scientific™, Waltham, MA, USA, Catalog #: M1661) as per the manufacturer’s protocol. A T100™ Thermal Cycler (Bio-Rad™, Hercules, CA, USA, Catalog #: 1861096) was used to run the reaction. 90 μL of Invitrogen™ RT-PCR Grade Water (Invitrogen™, Catalog #: AM9935) was then added to each sample. The synthesized cDNA was then placed into single-use aliquots and stored at −80 °C.

### 2.7. Quantitative Polymerase Chain Reaction (qPCR)

qPCR was performed using the Applied Biosystems™ PowerUp™ SYBR™ Green Master Mix (Applied Biosystems™, Foster City, CA, USA, Catalog #: A25918) as per the manufacturer’s protocol. The primers used were purchased from Sigma-Aldrich™ (Easy Oligo) and arrived pre-diluted in deionized water at a concentration of 100 µM. The primers were made into single-use aliquots upon arrival and stored at −20 °C. The primers used have the following sequences:

PRMT5 Forward Primer: 5′ GTTCTGCTATTCATAACCCCA 3′
PRMT5 Reverse Primer: 5′ AATCCAGCACTAATTCCTCA 3′
GAPDH Forward Primer: 5′ CTTTTGCGTCGCCAG 3′
GAPDH Reverse Primer: 5′ TTGATGGCAACAATATCCAC 3′

Primers were prepared for qPCR by adding 5 µL of reverse primer, 5 µL of forward primer, and 90 µL of Invitrogen™ RT-PCR Grade Water to a 0.6 mL microcentrifuge tube (5 µM final concentration of each primer). A total of 2 µL of the prepared 5 µM primer mix, 2 µL of the synthesized cDNA, 10 µL of the Applied Biosystems™ PowerUp™ SYBR™ Green Master Mix, and 6 µL of Invitrogen™ RT-PCR Grade Water were added to each well of a qPCR plate (final reaction mix contained a 500 nM concentration of each primer and 18 ng of cDNA assuming a 1:1 RNA to cDNA synthesis ratio). The qPCR plate was then centrifuged for 10 s using a Fisherbrand™ Mini Plate Spinner Centrifuge (Fisherbrand™, Pittsburgh, PA, USA, Catalog #: 14-100-143). An Applied Biosystems™ 7300 Real-Time PCR System was used to run the qPCR. All reactions were prepared in triplicates.

Data analysis was performed by the delta-delta-ct method (ΔΔCt). Delta ct was first calculated by subtracting each cell line’s average GAPDH ct value from its average PRMT5 ct value. Delta-delta ct was then calculated by subtracting the delta ct values of the control normal colon epithelial CCD 841 CoN cell line from the delta ct values of the CRC cell lines. The delta-delta ct values were then converted to fold values.

### 2.8. Protein Extraction

HCT116, SW620, Caco-2, HT-29, and CCD 841 CoN cells were cultured until 70% confluency was reached. The cells were then trypsinized and subsequently spun down into cell pellets. Total protein was then extracted from the cell pellets by the following freeze-thaw protocol. First, freeze-thaw lysis buffer (600 mM KCl, 20 mM Tris-Cl (pH 7.8), 20% Glycerol) was prepared [33]. Next, 10 µL of Thermo Scientific™ Halt™ Protease and Phosphatase Inhibitor Cocktail EDTA-free (100X) (Thermo Scientific™, Catalog #: 78445) was added to a microcentrifuge tube per 1 mL of freeze-thaw lysis buffer. Then, each cell pellet was suspended in 250 µL of freeze-thaw lysis buffer with protease and phosphatase inhibitor. The cell pellets were then placed in liquid nitrogen for 10 s and then allowed to thaw on ice. The cell pellets were then vortexed. The freeze-thaw-vortex steps were then repeated for a total of five times. The cell lysates were then passed five times through 29-gauge insulin syringes (Exel International™, Redondo Beach, CA, USA, Catalog #: 26028). The cell pellets were then placed in a microcentrifuge at max speed for 10 min at 4 °C. The supernatants were then placed into single-use aliquots and stored at −80 °C.

### 2.9. Protein Quantification

The concentration of the extracted protein was quantified using an Invitrogen™ Qubit™ Protein Assay Kit (Invitrogen™, Catalog #: Q33212), as per the manufacturer’s protocol.

### 2.10. Western Blot Analysis

Western blot was performed using the Invitrogen™ Western Devices Benchtop Bundle (Invitrogen™, Catalog #: IW3000S) as per the manufacturer’s protocol. The primary antibody used for PRMT5 detection was the Invitrogen™ PRMT5 Recombinant Rabbit Monoclonal Antibody (ST51-06) (Invitrogen™, Catalog #: MA5-32160) at a 1:1000 dilution. The secondary antibody used for PRMT5 detection was the Invitrogen™ Goat anti-Rabbit IgG (H+L) Secondary Antibody, HRP (Invitrogen™, Catalog #: 31460) at a 1:5000 dilution. Beta-actin at a 1:5000 dilution, as well as the Invitrogen™ No-Stain™ Protein Labeling Reagent (Invitrogen™, Catalog #: A44449), were used for normalization as per the manufacturer’s protocol. A Bio-Rad™ ChemiDoc™ MP Imaging System was used for blot imaging. The band intensities were quantified using Bio-Rad’s™ Image Lab software. Bio-Rad’s™ Image Lab software was also used for normalization by normalizing the quantified intensity of each band to the quantified intensity of its respective housekeeping protein (beta-actin) or total protein. Data analysis was performed by dividing the normalized band intensities of the CRC cell lines by the normalized band intensity of the control normal colon epithelial CCD 841 CoN cell line to arrive at the fold changes.

### 2.11. Cell Viability Assay

HCT116, SW620, HKe3, and LIM2405 cells were cultured until 70% confluency. The cells were then trypsinized and subsequently counted using the Countess™ II Automated Cell Counter (Invitrogen™, Catalog #: AMQAX1000) with Trypan Blue solution (Sigma-Aldrich™, Catalog #: T8154) as per the manufacturer’s protocol. The cells were then diluted to a concentration of 1.0 × 10^4^ cells/mL. 180 µL of Phosphate Buffered Saline (PBS) (Lonza™, Catalog #: 17-516Q) was then pipetted into all of the 36 outer wells of a BrandTech® cellGrade™ 96-well plate (BrandTech^®^, Essex, CT, USA, Catalog #: 781968) using a PIPETMAN M Multichannel P12x1200M (Gilson™, Middleton, WI, USA, Catalog #: F81021) pipette to avoid issues of edge effect evaporation. A total of 90 µL of the diluted cell solution (900 total cells) was then pipetted into the inner wells of the 96-well plate using the multichannel pipette. A total of 90 µL of MEM was then pipetted into one empty column of the 96-well plate. The 96-well plates were then placed in an incubator with an atmosphere of 5% CO_2_ at 37 °C for 24 h. 

Twenty-four hours later, 90 µL of MEM containing dimethyl sulfoxide (DMSO) (ATCC^®^, Catalog #: ATCC^®^ 4-X™) (Control cells) or PRMT5 inhibitor (EPZ015666) (Sigma-Aldrich™, Catalog #: SML1421) (Treated cells) was added to the 96-well plates using the multichannel pipette. The final concentrations of the PRMT5 inhibitor (and DMSO) in the 180 µL of MEM for the treated cells were 1 µM (with 0.00192% DMSO) and 10 µM (with 0.0192% DMSO). The final concentration of the DMSO in the 180 µL of MEM for the control cells was 0.0192% DMSO. A total of 90 µL of MEM was then pipetted into the one column containing only MEM in the 96-well plate. The 96-well plates were then placed in an incubator with an atmosphere of 5% CO_2_ at 37 °C for 60 h.

Sixty hours later, 20 µL of Invitrogen™ PrestoBlue™ HS Cell Viability Reagent (Invitrogen™, Catalog #: P50200) was then added to the inner wells of the 96-well plates using a PIPETMAN M Multichannel P12x200M (Gilson™, Catalog #: F81030) pipette. The 96-well plates were then gently mixed using a Scilogex Analog MX-M Microplate Mixer (Scilogex™, Rocky Hill, CT, USA, Catalog #: 822000049999). The 96-well plates were then placed in an incubator with an atmosphere of 5% CO_2_ at 37 °C for 2 h.

Two hours later, fluorescence readings were taken of the 96-well plates using a Beckman Coulter DTX 880 Multimode Detector plate reader at fluorescence excitation 535 nm and emission 595 nm wavelengths as per the manufacturer’s protocol.

Data analysis was performed by first calculating the mean relative fluorescence unit for each experimental data point. The mean relative fluorescence unit of the MEM-only wells was then subtracted from the mean relative fluorescence unit of the experimental wells to correct for background fluorescence. The percent change between the control and treated cells was then calculated. All reactions were prepared in sextuplets using all six inner wells per column for every experimental data point.

The PrestoBlue™ HS Cell Viability Reagent was chosen for our cell viability assay as PrestoBlue™ is a resazurin-based solution that uses the reducing power of cells to quantitatively measure the proliferation of cells. As such, our experiment determines the cell viability of cells by measuring the cells’ rates of proliferation [34].

The EPZ015666 PRMT5 inhibitor was chosen for our study as a previous study by Elayne Chan-Penebre et al. has shown that EPZ015666 is a potent and highly selective inhibitor of PRMT5 with an over 10,000-fold specificity against PRMT5 relative to other methyltransferases [35]. The PRMT5 inhibitor solution was prepared by dissolving 5 mg of EPZ015666 in 250 µL of DMSO to make a 20 mg/mL solution. This solution was then placed into single-use aliquots and stored at −80 °C. Serial dilutions of the EPZ01566 aliquots were then performed in MEM before treatment to arrive at final 20 µM and 2 µM solutions which were then added 1:1 (90 µL of treated MEM was added to the 90 µL of plated MEM) to arrive at the final treatment concentrations of 10 µM and 1 µM.

PRMT5 inhibitor concentrations of 1 µM and 10 µM were chosen for our study as our preliminary high-throughput cell viability assay results showed that PRMT5 inhibitor concentrations of 1 nM, 10 nM, and 100 nM did not show any substantial growth inhibition, while on the other hand, the PRMT5 inhibitor concentrations of 1 µM and 10 µM did indeed show significant growth inhibition. The results of our preliminary high-throughput cell viability assay can be seen in Table S1.

While many standard cell viability protocols call for plating 5000 cells in each well of the 96-well plate, we followed the recommendations of Thermo Fisher Scientific™ and ATCC^®^ to experimentally determine the optimal cell count and incubation period for our cell lines. This was done by plating serial dilutions of 7200, 3600, 1800, 900, and 450 cells in 90 µL of MEM into the inner wells of a 96-well plate. Twenty-four hours later, 90 µL of MEM was added to the inner wells of the 96-well plate. Sixty hours later, 20 µL of PrestoBlue™ was added to the inner wells of the 96-well plate, and then fluorescence readings were taken of the plates after 30 min, 1-h, and 2-h incubations. A graph was then generated of Relative Fluorescence Units vs. Cells per well. Cell counts of 450, 900, and in some cases, 1800, were determined to fall within the linear portion of the graph. Values for the 7200, 3600, and in some cases 1800 cells per well were seen to fall outside of the linear part of the line due to saturation of the PrestoBlue™ reagent, causing a plateau effect. As such, 900 cells per well was determined to be the ideal cell plating density for our experiment. Similarly, a 2-h incubation time post the addition of PrestoBlue™ was seen to produce the greatest linear results, and so a 2-h incubation time was chosen for our experiment.

### 2.12. Annexin V Assay

HCT116, SW620, HKe3, and LIM2405 cells were cultured until 70% confluency was reached. The cells were then trypsinized and subsequently counted using the Countess™ II Automated Cell Counter with Trypan Blue solution as per the manufacturer’s protocol. The cells were then diluted to a concentration of 3.0 × 10^4^ cells/mL. A total of 1 mL of the diluted cell solution (30,000 total cells) was then pipetted into the wells of a Denville^®^ 6-well Cell Culture Plate (Denville^®^, Holliston, MA, USA, Catalog #: 1156D98). The 6-well plates were then placed in an incubator with an atmosphere of 5% CO_2_ at 37 °C for 24 h.

Twenty-four hours later, 1 mL of MEM containing DMSO (Control cells) or PRMT5 inhibitor (Treated cells) was added to the 6-well plates. The final concentration of the PRMT5 inhibitor (and DMSO) in the 2 mL of MEM for the treated cells was 10 µM (with 0.0192% DMSO). The final concentration of the DMSO in the 2 mL of MEM for the control cells was 0.0192% DMSO. The 6-well plates were then placed in an incubator with an atmosphere of 5% CO_2_ at 37 °C for 60 h.

Sixty hours later, an annexin assay was then performed using the Guava^®^ Annexin Red Kit (Guava^®^, Hayward, CA, USA, Catalog #: FCCH100108) as per the manufacturer’s protocol. The samples were run in a Guava^®^ EasyCyte™ mini. Data analysis was performed by subtracting the percent of Annexin V+ cells in the control sample from the percent of Annexin V+ cells in the treated sample to arrive at ΔAnnexin V+ cells.

### 2.13. Cell Cycle Assay

HCT116, SW620, HKe3, and LIM2405 cells were cultured until 70% confluency. The cells were then trypsinized and subsequently counted using the Countess™ II Automated Cell Counter with Trypan Blue solution as per the manufacturer’s protocol. The cells were then diluted to a concentration of 3.0 × 10^4^ cells/mL. 1 mL of the diluted cell solution (30,000 total cells) was then pipetted into the wells of a Denville^®^ 6-well Cell Culture Plate. The 6-well plates were then placed in an incubator with an atmosphere of 5% CO_2_ at 37 °C for 24 h.

Twenty-four hours later, 1 mL of MEM containing DMSO (Control cells) or PRMT5 inhibitor (Treated cells) was added to the 6-well plates. The final concentration of the PRMT5 inhibitor (and DMSO) in the 2 mL of MEM for the treated cells was 10 µM (with 0.0192% DMSO). The final concentration of the DMSO in the 2 mL of MEM for the control cells was 0.0192% DMSO. The 6-well plates were then placed in an incubator with an atmosphere of 5% CO_2_ at 37 °C for 60 h.

Sixty hours later, a cell cycle assay was then performed using the Guava^®^ Cell Cycle Reagent (Guava^®^, Catalog #: 4500-0220) as per the manufacturer’s protocol. The samples were run in a Guava^®^ EasyCyte™ mini. Data analysis was performed by subtracting the percent of G_1_, S, & G_2_ phase cells in the control sample from the percent of G_1_, S, & G_2_ phase cells in the treated sample to arrive at ΔG_1_, ΔS, & ΔG_2_ phase cells.

### 2.14. Statistical Analysis

Statistical analysis was performed using Microsoft™ Office Excel. A two-tailed *t*-test was used to determine statistical significance (*p* < 0.05). Due to the severe misleading effects that outlier data points can have on data analysis as shown by Osborne JW, outlier data points were determined and removed from data analysis, according to Iglewicz and Hoaglin’s outlier test with modified z-scores using the recommended outlier criterion of modified Z score ≥ 3.5 [36,37].

## 3. Results

### 3.1. PRMT5 Expression Is Upregulated, as Well as Positively Correlated with KRAS Expression, in CRC Patient Datasets

**Figure 1:**
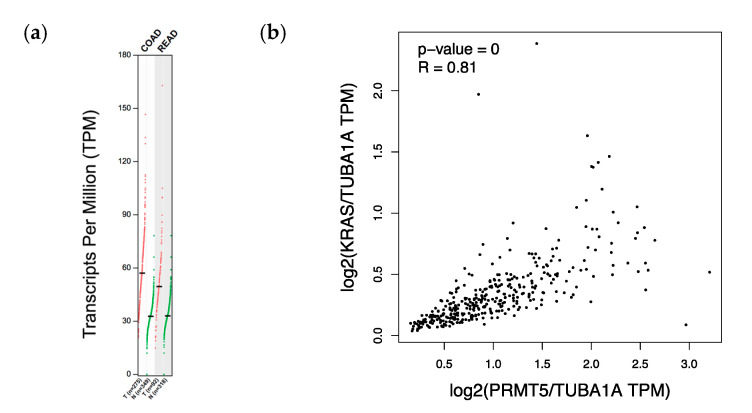
PRMT5 expression is shown to be upregulated, as well as positively correlated with *KRAS* expression, in CRC patient datasets. (**a**) RNA-Seq data of CRC patients from the TCGA database using the GEPIA website showing that PRMT5 is overexpressed (*q* < 0.01) in CRC patient tumor samples when compared to normal colon and rectum tissue; (**b**) RNA-Seq data of CRC patients from the TCGA database using the GEPIA website displaying that PRMT5 expression is strongly positively correlated (*p* < 0.005, R = 0.81) with *KRAS* expression in CRC patient tumor samples.

While PRMT5 has previously been shown to be overexpressed in CRC cells when compared to normal colonic mucosal FHC cells, we sought to determine whether PRMT5 is overexpressed in CRC patient datasets [27]. Upon analyzing the RNA-Seq data of CRC patients from the TCGA database using the GEPIA website, we found that PRMT5 is indeed overexpressed (*q* < 0.01) in CRC patient tumor samples when compared to normal colon and rectum tissue. We next sought to determine whether PRMT5 expression is correlated with *KRAS* expression in CRC patient datasets. After examining the RNA-Seq data of CRC patients from the TCGA database using the GEPIA website, we found that PRMT5 expression is in fact strongly positively correlated (*p* < 0.005, R = 0.81) with *KRAS* expression in CRC patient tumor samples (Figure 1).

### 3.2. PRMT5 mRNA Is Further Overexpressed in KRAS Mutant CRC Cells

**Figure 2:**
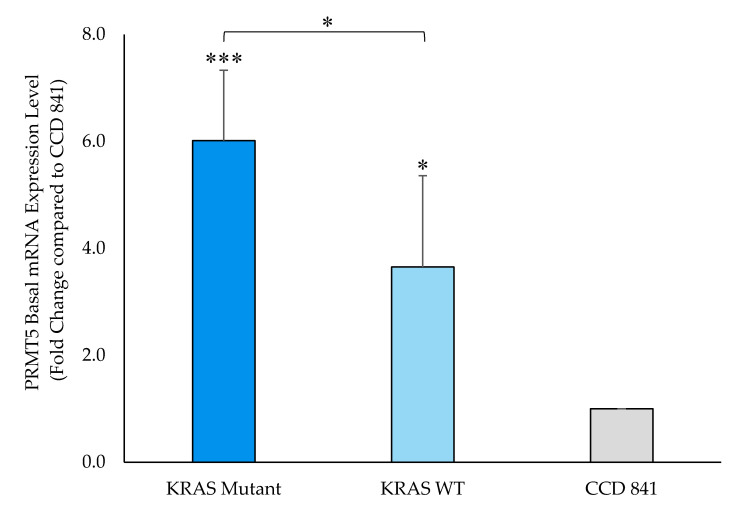
PRMT5 is shown to be further overexpressed in the *KRAS* mutant CRC cells at the transcriptional level by qPCR analysis. Quantified qPCR assay results show that PRMT5 mRNA is 1.6-Fold (*p* < 0.05) further overexpressed in the *KRAS* mutant CRC cells when compared to the *KRAS* WT CRC cells. Data are expressed as means + SD from three independent experiments using four CRC cell lines (2 *KRAS* mutant, 2 *KRAS* WT), as well as one normal colon cell line. Data for the individual cell lines can be seen in Figures S2 and S3. * represents *p* < 0.05; *** represents *p* < 0.005.

Having demonstrated that PRMT5 expression is upregulated, as well as positively correlated with *KRAS* expression, in CRC patient datasets, we next sought to determine whether PRMT5 is further overexpressed in *KRAS* mutant CRC cells when compared to *KRAS* WT CRC cells. Our qPCR assay results demonstrate that while PRMT5 mRNA is 3.7-Fold (*p* < 0.05) overexpressed in *KRAS* WT CRC cells when compared to normal CCD 841 CoN colon cells, the *KRAS* mutant CRC cells show a more substantial 6.0-Fold (*p* < 0.005) overexpression of PRMT5 mRNA. More significantly, our qPCR assay results display that when comparing the PRMT5 expression of the *KRAS* mutant CRC cells to the *KRAS* WT CRC cells, the *KRAS* mutant CRC cells show a further 1.6-Fold (*p* < 0.05) overexpression of PRMT5 at the transcriptional level (Figure 2).

### 3.3. PRMT5 Protein Is Further Overexpressed in KRAS Mutant CRC Cells

**Figure 3:**
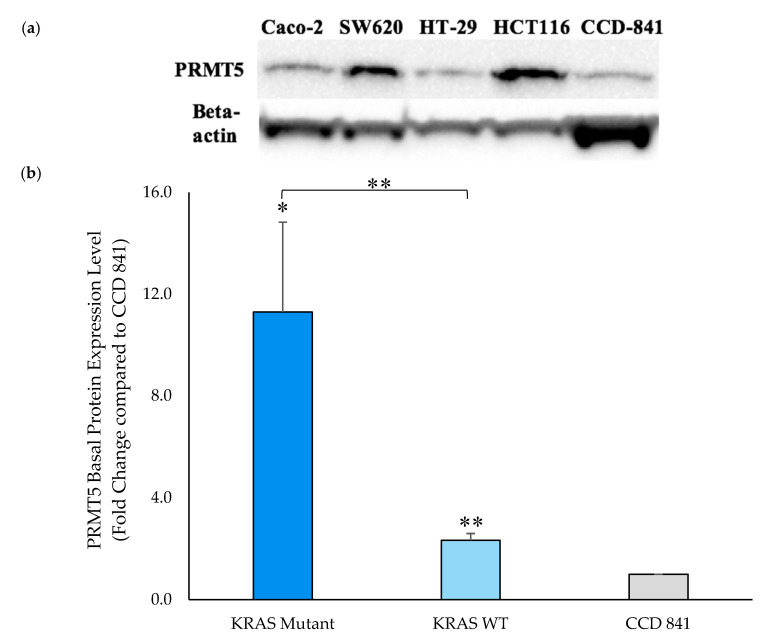
PRMT5 is shown to be further overexpressed in the *KRAS* mutant CRC cells at the translational level by Western blot analysis. (**a**) Western blot assay results displaying PRMT5 and corresponding beta-actin bands; (**b**) Quantified Western blot assay results showing that PRMT5 protein is 4.8-Fold (*p* < 0.01) further overexpressed in the *KRAS* mutant CRC cells when compared to the *KRAS* WT CRC cells. Data are expressed as means + SD from two independent experiments using four CRC cell lines (2 *KRAS* mutant, 2 *KRAS* WT), as well as one normal colon cell line. The uncropped Western blot membranes can be seen in Figure S1. Data for the individual cell lines can be seen in Figures S2 and S3. * represents *p* < 0.05; ** represents *p* < 0.01.

Having shown that PRMT5 is further overexpressed at the transcriptional level in the *KRAS* mutant CRC cells when compared to the *KRAS* WT CRC cells, a Western blot assay was then performed to ascertain whether this overexpression of PRMT5 at the transcriptional level affected a further overexpression of PRMT5 at the translational level in the *KRAS* mutant CRC cells. Our Western blot assay results demonstrate that while PRMT5 protein is 2.3-Fold (*p* < 0.01) overexpressed in the *KRAS* WT CRC cells when compared to normal CCD 841 CoN colon cells, the *KRAS* mutant CRC cells show a more substantial 11.3-Fold (*p* < 0.05) overexpression of PRMT5 protein. More significantly, our Western blot assay results display that when comparing the PRMT5 expression of the *KRAS* mutant CRC cells to the *KRAS* WT CRC cells, the *KRAS* mutant CRC cells show a further 4.8-Fold (*p* < 0.01) overexpression of PRMT5 at the translational level (Figure 3). 

### 3.4. PRMT5 Inhibition Lowers Cell Viability Further in KRAS Mutant CRC Cells

**Figure 4:**
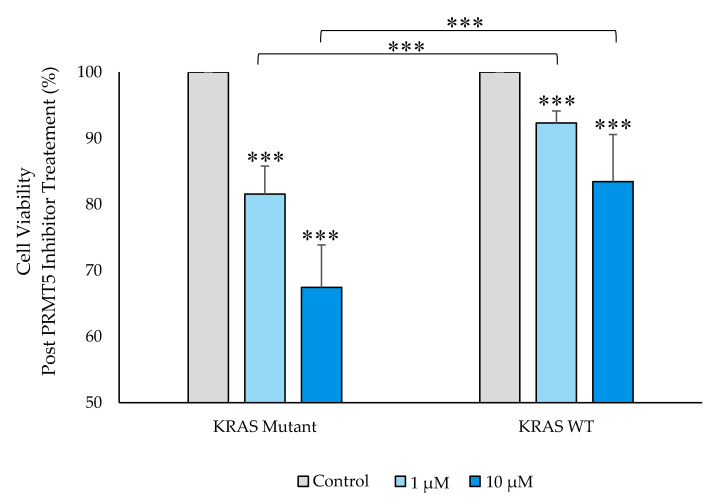
PRMT5 inhibitor treatment is shown to further decrease cell viability in the *KRAS* mutant CRC cells by cell viability analysis. Quantified cell viability assay results show that PRMT5 inhibitor treatment affected a further 2.4-fold (*p* < 0.005) and 2.0-fold (*p* < 0.005) decrease in cell viability after 60 h of treatment with 1 µM & 10 µM PRMT5 inhibitor concentrations, respectively, in the *KRAS* mutant CRC cells when compared to the *KRAS* WT CRC cells. Data are expressed as means + SD from three independent experiments using four CRC cell lines (2 *KRAS* mutant, 2 *KRAS* WT). Data for the individual cell lines can be seen in Figures S2 and S3. *** represents *p* < 0.005.

Having ascertained that PRMT5 is further overexpressed at both the translational and transcriptional levels in the *KRAS* mutant CRC cells when compared to the *KRAS* WT CRC cells, a cell viability assay was then carried out to determine whether this further overexpression of PRMT5 in the *KRAS* mutant CRC cells has clinical applicability. The results of our cell viability assay indicate that while the *KRAS* WT CRC cells show 7.7% (*p* < 0.005) and 16.5% (*p* < 0.01) decreases in cell viability, after 60 h of treatment with 1 µM & 10 µM concentrations of PRMT5 inhibitor, respectively, the *KRAS* mutant CRC cells show more substantial 18.4% (*p* < 0.005) and 32.6% (*p* < 0.005) decreases in cell viability, respectively. More significantly, our cell viability results demonstrate that the further overexpression of PRMT5 in the *KRAS* mutant CRC cells affected a further 2.4-fold (*p* < 0.005) and 2.0-fold (*p* < 0.005) decrease in cell viability after 60 h of treatment with 1 µM & 10 µM PRMT5 inhibitor concentrations, respectively, when compared to the *KRAS* WT CRC cells (Figure 4). 

### 3.5. PRMT5 Inhibition Stimulates Apoptosis in KRAS Mutant CRC Cells

**Figure 5:**
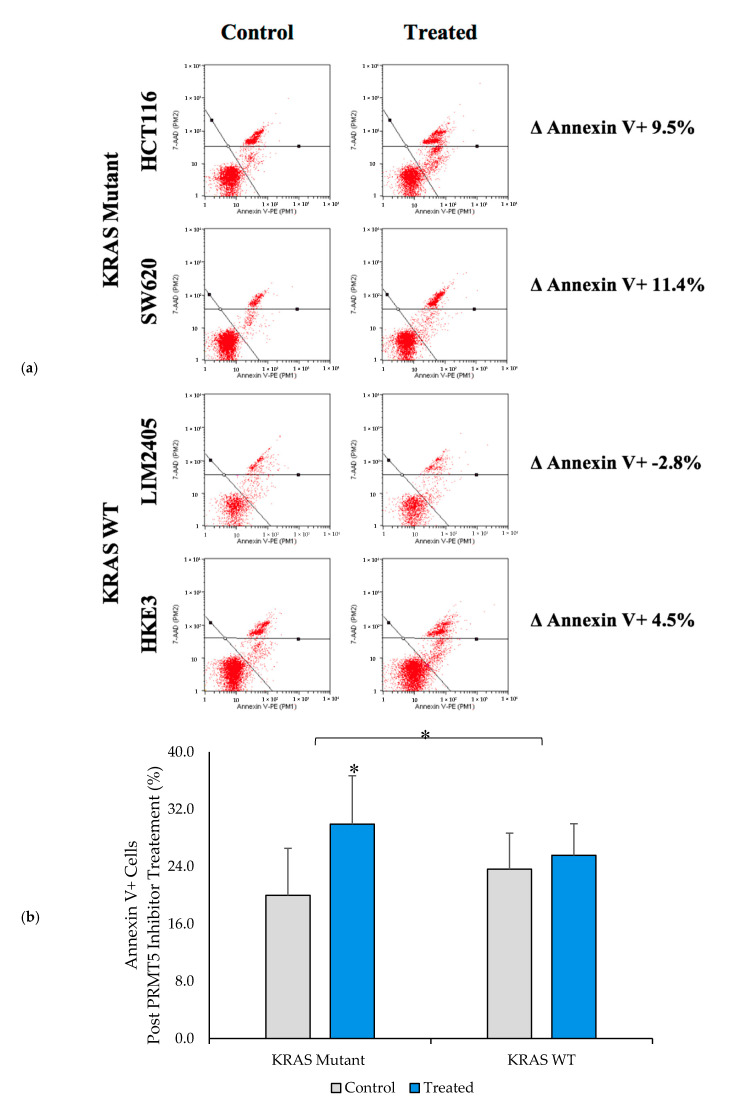
PRMT5 inhibitor treatment is shown to affect apoptosis in the *KRAS* mutant CRC cells by Annexin V analysis. (**a**) Flow cytometry dot plots displaying Annexin V assay results; (**b**) Quantified Annexin V assay results displaying that PRMT5 inhibitor treatment shows an 8.0% (*p* < 0.05) greater increase in apoptosis after 60 h of 10 µM PRMT5 inhibitor treatment in the *KRAS* mutant CRC cells when compared to the *KRAS* WT CRC cells. Data are expressed as means + SD from three independent experiments using four CRC cell lines (2 *KRAS* mutant, 2 *KRAS* WT). Data for the individual cell lines can be seen in Figures S2 and S3. * represents *p* < 0.05.

Having shown that PRMT5 inhibition leads to a further decrease in cell viability in the *KRAS* mutant CRC cells when compared to the *KRAS* WT CRC cells, we next sought to determine whether the *KRAS* mutant CRC cells would show a greater level of apoptosis after 60 h of 10 µM PRMT5 inhibitor treatment when compared to the *KRAS* WT CRC cells. The results of our Annexin V assay demonstrate that while the *KRAS* WT CRC cells show no significant increase in apoptosis (*p* > 0.05), after 60 h of 10 µM PRMT5 inhibitor treatment, the *KRAS* mutant CRC cells showed a significant 10.0% (*p* < 0.05) increase in apoptosis. More significantly, our Annexin V assay results display that the *KRAS* mutant CRC cells show an 8.0% (*p* < 0.05) greater increase in apoptosis after 60 h of 10 µM PRMT5 inhibitor treatment when compared to the *KRAS* WT CRC cells (Figure 5). 

### 3.6. PRMT5 Inhibition Triggers G_2_ Phase Cell Cycle Arrest in KRAS Mutant CRC Cells

**Figure 6:**
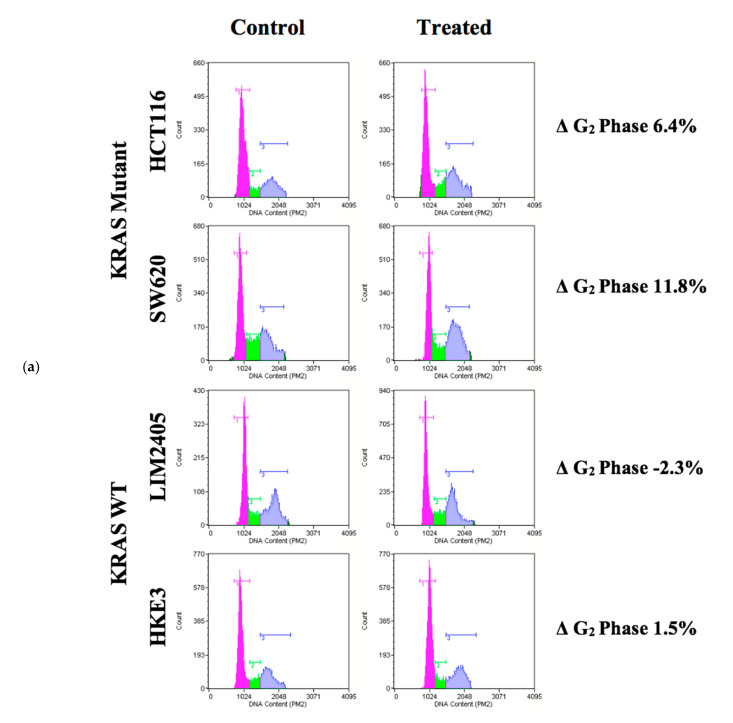

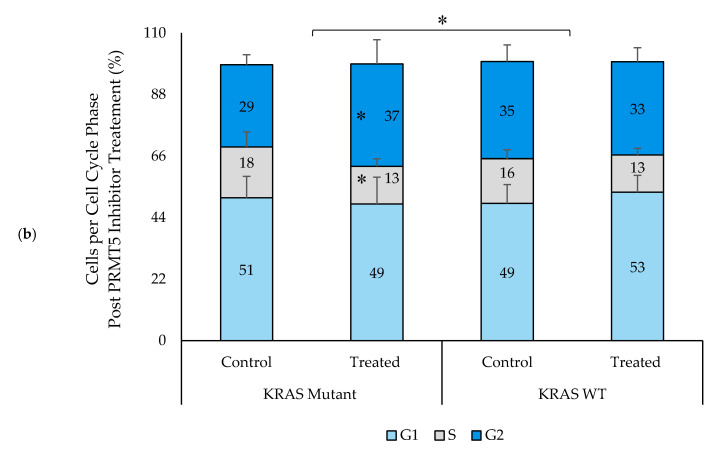
PRMT5 inhibitor treatment is shown to result in a significant G_2_ phase cell cycle arrest in the *KRAS* mutant CRC cells by cell cycle analysis. (**a**) Flow cytometry dot plots displaying cell cycle assay results; (**b**) Quantified cell cycle assay results displaying that PRMT5 inhibitor treatment showed an 8.6% (*p* < 0.005) greater increase in G_2_ phase cells, as well as 6.2% (*p* < 0.05) and 2.3% (*p* < 0.05) greater decreases in G_1_ and S phase cells respectively after 60 h of 10 µM PRMT5 inhibitor treatment in the *KRAS* mutant CRC cells when compared to the *KRAS* WT CRC cells. Data are expressed as means + SD from four independent experiments using four CRC cell lines (2 *KRAS* mutant, 2 *KRAS* WT). Data for the individual cell lines can be seen in Figures S2 and S3. * represents *p* < 0.05.

As several previous studies have shown that PRMT5 inhibition leads to cell cycle arrest, we next sought to determine whether the *KRAS* mutant CRC cells would show a greater rate of cell cycle arrest after 60 h of 10 µM PRMT5 inhibitor treatment when compared to the *KRAS* WT CRC cells [26,38]. The results of our cell cycle assay demonstrate that while the *KRAS* WT CRC cells show no significant differences in the number of G_1_, S, or G_2_ phase cells (*p* > 0.05), after 60 h of 10 µM PRMT5 inhibitor treatment, the *KRAS* mutant CRC cells showed a significant 7.3% (*p* < 0.05) increase in G_2_ phase cells, as well as a 5.0% (*p* < 0.05) decrease in S phase cells. More significantly, our cell cycle assay results display that the *KRAS* mutant CRC cells show an 8.6% (*p* < 0.005) greater increase in G_2_ phase cells, as well as 6.2% (*p* < 0.05) and 2.3% (*p* < 0.05) greater decreases in G_1_ and S phase cells, respectively, after 60 h of 10 µM PRMT5 inhibitor treatment when compared to the *KRAS* WT CRC cells (Figure 6).

## 4. Discussion

PRMT5 is a transcription regulator for multiple cellular processes including gene expression, protein modification, signal transduction, as well as cell cycle progression [24,26]. PRMT5 has been previously shown to be overexpressed in approximately 75% of CRC patient tumor samples, as well as negatively correlated with CRC patient survival [27]. PRMT5 inhibition and knockdown have also been seen to result in reduced rates of cellular proliferation, as well as increased rates of apoptosis and cell cycle arrest in both in vitro and in vivo studies [26,27]. As nearly 45% of CRC patients harbor a *KRAS* mutation, for which no targeted therapy is currently available, we performed this study to determine whether PRMT5 can act as a surrogate target for mutated *KRAS* in CRC [13].

While previous studies have shown that PRMT5 is overexpressed in CRC cells in general, our results revealed that PRMT5 expression is upregulated, as well as positively correlated with *KRAS* expression, in CRC patient datasets. Moreover, our qPCR and Western blot assay results further showed that when comparing the PRMT5 expression of the *KRAS* mutant CRC cells to the *KRAS* WT CRC cells, the *KRAS* mutant CRC cells showed a further overexpression of PRMT5 at both the transcriptional and translational levels [27]. These results are significant as they indicate that the expression level of PRMT5 is correlated with the mutation status of *KRAS*. In light of these findings, we therefore hypothesized that PRMT5 inhibition may show greater effects in the *KRAS* mutant CRC cells when compared to the *KRAS* WT CRC cells.

Previous studies have shown that PRMT5 inhibition leads to a decrease in cellular proliferation [26,27]. Our cell viability assay results further showed that PRMT5 inhibition displayed a substantially greater reduction in cell viability in the *KRAS* mutant CRC cells when compared to the *KRAS* WT CRC cells. Similarly, our annexin V assay results showcased that the *KRAS* mutant CRC cells underwent a significantly greater degree of apoptosis when compared to the *KRAS* WT CRC cells, following treatment with PRMT5 inhibitor. These findings are significant as they indicate that a PRMT5 inhibiting treatment may prove to be an effective therapy for *KRAS* mutant CRC.

While previous studies have shown that PRMT5 inhibition leads to cell cycle arrest, the results of our cell cycle assay further show that following treatment with PRMT5 inhibitor, the *KRAS* mutant CRC cells showed a much greater level of G_2_ phase cell cycle arrest when compared to the *KRAS* WT CRC cells [26,38]. It’s interesting to note that while our results agree with several previous studies that also found G_2_ phase cell cycle arrest following treatment with EPZ015666 PRMT5 inhibitor, some previous studies have found that PRMT5 knockdown is associated with a G_1_ phase cell cycle arrest [18,26,27,38]. The mode at which PRMT5 is targeted is likely the determinant here, and further study is needed to fully elucidate this phenomenon. 

Further research is currently underway to determine how exactly PRMT5 and *KRAS* interact, as well as the mechanism behind PRMT5 upregulation. Several previous studies have identified *FGFR3* and *eIF4E* as two key genes that are regulated by PRMT5 [27,39,40]. Both *FGFR3* and *eIF4E* have previously been shown to be overexpressed in several types of cancers, including CRC, and they have both also been found to promote tumor growth [41,42,43,44]. Additionally, previous studies have shown that upon PRMT5 knockdown, *FGFR3* and *eIF4E* expression were found to be significantly lower in the si-PRMT5 CRC cell lines when compared to the si-NC CRC cell lines [27,39,40]. While the exact molecular mechanisms through which PRMT5 acts on *FGFR3* and *eIF4E* have already been demonstrated, the precise molecular details explaining how *KRAS* is involved in this pathway is still being determined [27,40]. Any information found regarding the exact molecular mechanism by which PRMT5 and *KRAS* crosstalk can be therapeutically utilized towards developing new effective treatments for *KRAS* mutant CRC patients.

## 5. Conclusions

In conclusion, our research strongly supports that a PRMT5 inhibiting treatment may prove to be an effective therapy for *KRAS* mutant CRC. Our results show that PRMT5 expression is upregulated, as well as positively correlated with *KRAS* expression, in CRC patient datasets. Additionally, our data demonstrate that PRMT5 is further overexpressed in the *KRAS* mutant CRC cells when compared to the *KRAS* WT CRC cells at both the transcriptional and translational levels. Moreover, PRMT5 inhibition resulted in a further decrease in cell viability, as well as a further increase in apoptosis and G_2_ phase cell cycle arrest in the *KRAS* mutant CRC cells when compared to the *KRAS* WT CRC cells. Our research therefore indicates that PRMT5 and *KRAS* may crosstalk, and thus, PRMT5 can potentially be used as a surrogate target for mutated *KRAS* in CRC.

## Supplementary Materials

The following are available online at https://www.mdpi.com/2072-6694/12/8/2091/s1, Figure S1: Uncropped Western blot membranes, Figure S2: Experimental data for isogenic cell lines HCT116 (*KRAS* mutant) and HKE3 (*KRAS* WT), Figure S3: Experimental data for the individual cell lines used in Figure 2, Figure 3, Figure 4, Figure 5 and Figure 6, Table S1: Percent inhibition of CRC cell lines after treatment with several concentrations of PRMT5 inhibitor.

## References

[1] Siegel R.L., Miller K.D., Goding Sauer A., Fedewa S.A., Butterly L.F., Anderson J.C., Cercek A., Smith R.A., Jemal A. (2020). Colorectal cancer statistics, 2020. CA Cancer J. Clin..

[2] Jass J.R. (2007). Classification of colorectal cancer based on correlation of clinical, morphological and molecular features. Histopathology.

[3] Tanaka T. (2009). Colorectal carcinogenesis: Review of human and experimental animal studies. J. Carcinog..

[4] Fearon E.R. (2011). Molecular genetics of colorectal cancer. Annu. Rev. Pathol..

[5] Wee P., Wang Z. (2017). Epidermal growth factor receptor cell proliferation signaling pathways. Cancers.

[6] Jhawer M., Goel S., Wilson A.J., Montagna C., Ling Y.H., Byun D.S., Nasser S., Arango D., Shin J., Klampfer L. (2008). PIK3CA mutation/PTEN expression status predicts response of colon cancer cells to the epidermal growth factor receptor inhibitor cetuximab. Cancer Res..

[7] Zhao B., Wang L., Qiu H., Zhang M., Sun L., Peng P., Yu Q., Yuan X. (2017). Mechanisms of resistance to anti-EGFR therapy in colorectal cancer. Oncotarget.

[8] Brand T.M., Wheeler D.L. (2012). KRAS mutant colorectal tumors: Past and present. Small GTPases.

[9] Uhlyarik A., Piurko V., Papai Z., Raso E., Lahm E., Kiss E., Sikter M., Vachaja J., Kenessey I., Timar J. (2020). EGFR protein expression in KRAS wild-type metastatic colorectal cancer is another negative predictive factor of the cetuximab therapy. Cancers.

[10] Giehl K. (2005). Oncogenic Ras in tumour progression and metastasis. Biol. Chem..

[11] Pylayeva-Gupta Y., Grabocka E., Bar-Sagi D. (2011). RAS oncogenes: Weaving a tumorigenic web. Nat. Rev. Cancer.

[12] Downward J. (1998). Ras signalling and apoptosis. Curr. Opin. Genet. Dev..

[13] Bos J.L. (1989). Ras oncogenes in human cancer: A review. Cancer Res..

[14] Porru M., Pompili L., Caruso C., Biroccio A., Leonetti C. (2018). Targeting KRAS in metastatic colorectal cancer: Current strategies and emerging opportunities. J. Exp. Clin. Cancer Res..

[15] Dias Carvalho P., Machado A.L., Martins F., Seruca R., Velho S. (2019). Targeting the tumor microenvironment: an unexplored strategy for mutant KRAS tumors. Cancers.

[16] Blanc R.S., Richard S. (2017). Arginine Methylation: The Coming of Age. Mol. Cell.

[17] Andreu-Perez P., Esteve-Puig R., de Torre-Minguela C., Lopez-Fauqued M., Bech-Serra J.J., Tenbaum S., Garcia-Trevijano E.R., Canals F., Merlino G., Avila M.A. (2011). Protein arginine methyltransferase 5 regulates ERK1/2 signal transduction amplitude and cell fate through CRAF. Sci. Signal..

[18] Scoumanne A., Zhang J., Chen X. (2009). PRMT5 is required for cell-cycle progression and p53 tumor suppressor function. Nucleic Acids Res..

[19] Tewary S.K., Zheng Y.G., Ho M.C. (2019). Protein arginine methyltransferases: Insights into the enzyme structure and mechanism at the atomic level. Cell. Mol. Life Sci..

[20] Jiang H., Zhu Y., Zhou Z., Xu J., Jin S., Xu K., Zhang H., Sun Q., Wang J., Xu J. (2018). PRMT5 promotes cell proliferation by inhibiting BTG2 expression via the ERK signaling pathway in hepatocellular carcinoma. Cancer Med..

[21] Cho E.C., Zheng S., Munro S., Liu G., Carr S.M., Moehlenbrink J., Lu Y.C., Stimson L., Khan O., Konietzny R. (2012). Arginine methylation controls growth regulation by E2F-1. EMBO J..

[22] Li Y., Diehl J.A. (2015). PRMT5-dependent p53 escape in tumorigenesis. Oncoscience.

[23] Wei H., Wang B., Miyagi M., She Y., Gopalan B., Huang D.B., Ghosh G., Stark G.R., Lu T. (2013). PRMT5 dimethylates R30 of the p65 subunit to activate NF-kappaB. Proc. Natl. Acad. Sci. USA.

[24] Stopa N., Krebs J.E., Shechter D. (2015). The PRMT5 arginine Methyltransferase: Many roles in development, cancer and beyond. Cell. Mol. Life Sci..

[25] Zhang S., Ma Y., Hu X., Zheng Y., Chen X. (2019). Targeting PRMT5/Akt signalling axis prevents human lung cancer cell growth. J. Cell. Mol. Med..

[26] Vinet M., Suresh S., Maire V., Monchecourt C., Nemati F., Lesage L., Pierre F., Ye M., Lescure A., Brisson A. (2019). Protein arginine methyltransferase 5: A novel therapeutic target for triple-negative breast cancers. Cancer Med..

[27] Zhang B., Dong S., Zhu R., Hu C., Hou J., Li Y., Zhao Q., Shao X., Bu Q., Li H. (2015). Targeting protein arginine methyltransferase 5 inhibits colorectal cancer growth by decreasing arginine methylation of eIF4E and FGFR3. Oncotarget.

[28] RAS Cell Lines. https://www.cancer.gov/research/key-initiatives/ras/outreach/reference-reagents/cell-lines.

[29] Shirasawa S., Furuse M., Yokoyama N., Sasazuki T. (1993). Altered growth of human colon cancer cell lines disrupted at activated Ki-ras. Science.

[30] Adding Antibiotics or Antimycotics to Cell Culture Medium. https://www.atcc.org/Global/FAQs/2/1/Adding%20antibiotics%20or%20antimycotics%20to%20cell%20culture%20medium-79.aspx.

[31] Ryu A.H., Eckalbar W.L., Kreimer A., Yosef N., Ahituv N. (2017). Use antibiotics in cell culture with caution: Genome-wide identification of antibiotic-induced changes in gene expression and regulation. Sci. Rep..

[32] Tang Z., Li C., Kang B., Gao G., Li C., Zhang Z. (2017). GEPIA: A web server for cancer and normal gene expression profiling and interactive analyses. Nucleic Acids Res..

[33] Tansey W.P. (2006). Freeze-thaw lysis for extraction of proteins from Mammalian cells. CSH Protoc..

[34] PrestoBlue™ HS Cell Viability Reagent. https://www.thermofisher.com/order/catalog/product/P50201#/P50201.

[35] Chan-Penebre E., Kuplast K.G., Majer C.R., Boriack-Sjodin P.A., Wigle T.J., Johnston L.D., Rioux N., Munchhof M.J., Jin L., Jacques S.L. (2015). A selective inhibitor of PRMT5 with in vivo and in vitro potency in MCL models. Nat. Chem. Biol..

[36] Iglewicz B., Hoaglin D.C. (1993). How to Detect and Handle Outliers.

[37] Osborne J.W., Overbay A.M. (2004). The power of outliers (and why researchers should ALWAYS check for them). Pract. Assess. Res. Eval..

[38] Braun C.J., Stanciu M., Boutz P.L., Patterson J.C., Calligaris D., Higuchi F., Neupane R., Fenoglio S., Cahill D.P., Wakimoto H. (2017). Coordinated splicing of regulatory detained introns within oncogenic transcripts creates an exploitable vulnerability in malignant Glioma. Cancer Cell.

[39] Lim J.H., Lee Y.M., Lee G., Choi Y.J., Lim B.O., Kim Y.J., Choi D.K., Park J.W. (2014). PRMT5 is essential for the eIF4E-mediated 5’-cap dependent translation. Biochem. Biophys. Res. Commun..

[40] Gu Z., Gao S., Zhang F., Wang Z., Ma W., Davis R.E., Wang Z. (2012). Protein arginine methyltransferase 5 is essential for growth of lung cancer cells. Biochem. J..

[41] Fromme J.E., Schmitz K., Wachter A., Grzelinski M., Zielinski D., Koppel C., Conradi L.C., Homayounfar K., Hugo T., Hugo S. (2018). FGFR3 mRNA overexpression defines a subset of oligometastatic colorectal cancers with worse prognosis. Oncotarget.

[42] Xu T., Zong Y., Peng L., Kong S., Zhou M., Zou J., Liu J., Miao R., Sun X., Li L. (2016). Overexpression of eIF4E in colorectal cancer patients is associated with liver metastasis. Onco. Targets Ther..

[43] Sonvilla G., Allerstorfer S., Heinzle C., Stattner S., Karner J., Klimpfinger M., Wrba F., Fischer H., Gauglhofer C., Spiegl-Kreinecker S. (2010). Fibroblast growth factor receptor 3-IIIc mediates colorectal cancer growth and migration. Br. J. Cancer.

[44] Chao M.W., Wang L.T., Lai C.Y., Yang X.M., Cheng Y.W., Lee K.H., Pan S.L., Teng C.M. (2015). eIF4E binding protein 1 expression is associated with clinical survival outcomes in colorectal cancer. Oncotarget.

