# Supplementary material for: Protein Arginine Methyltransferase 5 as a Therapeutic Target for KRAS Mutated Colorectal Cancer
Source: Cancers (Basel). 2020 Jul 28;12(8):2091. doi: 10.3390/cancers12082091 (PMC7465151; doi:10.3390/cancers12082091)
Supplement: Supplementary file 1 [file cancers-12-02091-s001.pdf]

cancers

MDPI

# Supplementary Materials:

# Protein Arginine Methyltransferase 5 as a Therapeutic Target for *KRAS* Mutated Colorectal Cancer

**David Shifteh, Tzuriel Sapir, Moshe Pahmer, Adam Haimowitz, Sanjay Goel and Radhashree Maitra**

(a)

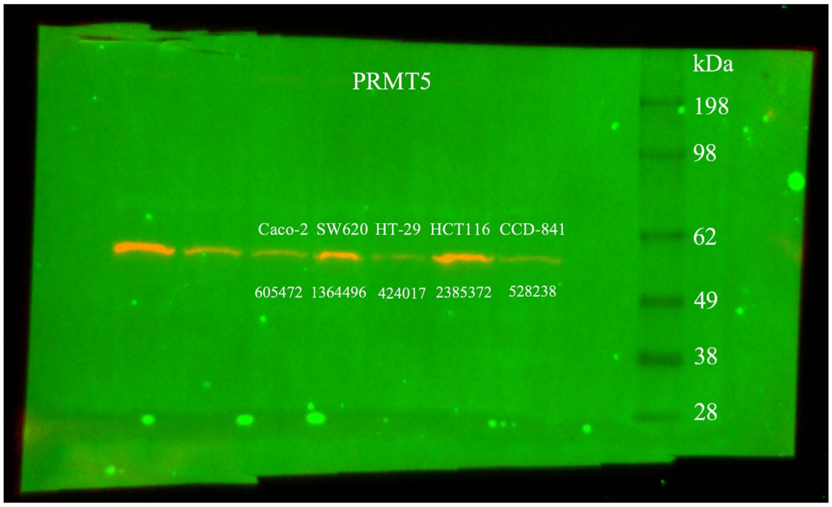


(b)

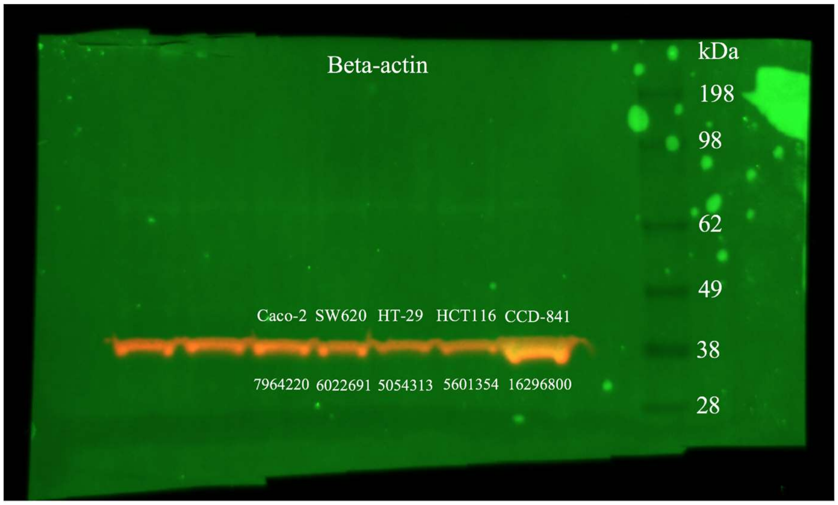

(c)

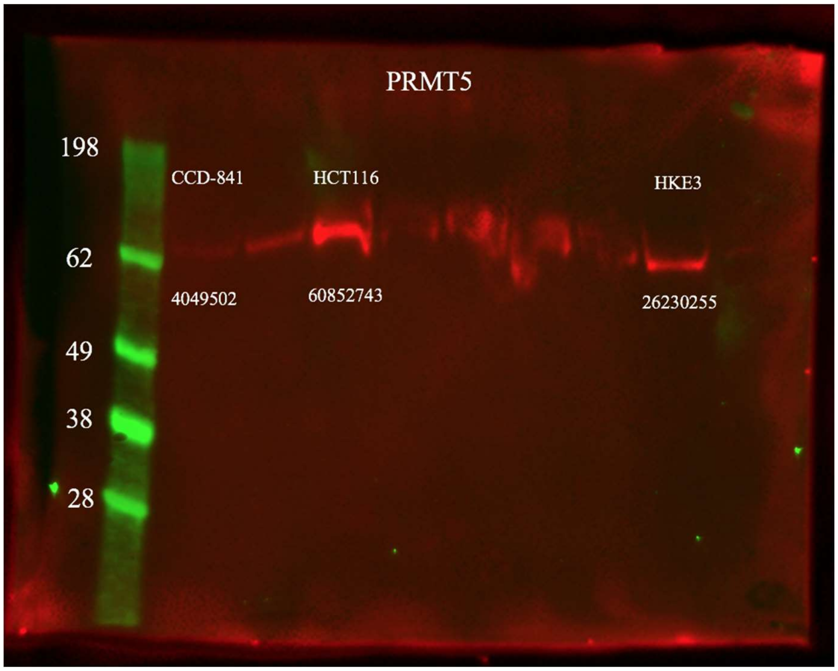


(d)

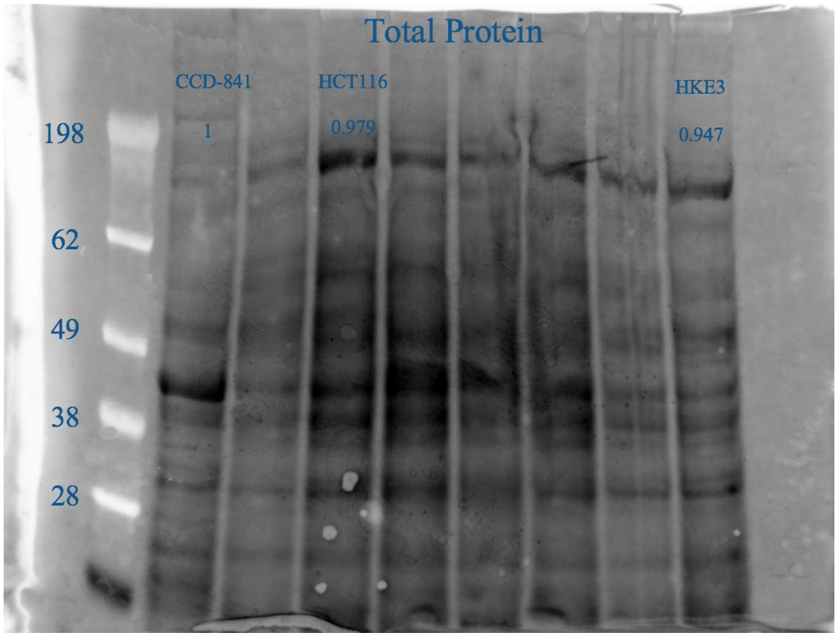


**Figure S1.** Uncropped western blot membranes. (**a**) Uncropped PRMT5 probed western blot membrane from Figure 3; (**b**) Uncropped beta-actin probed western blot membrane from Figure 3; (**c**) Uncropped PRMT5 probed western blot membrane from Figure S2; (**d**) Uncropped total protein western blot membrane from Figure S2.

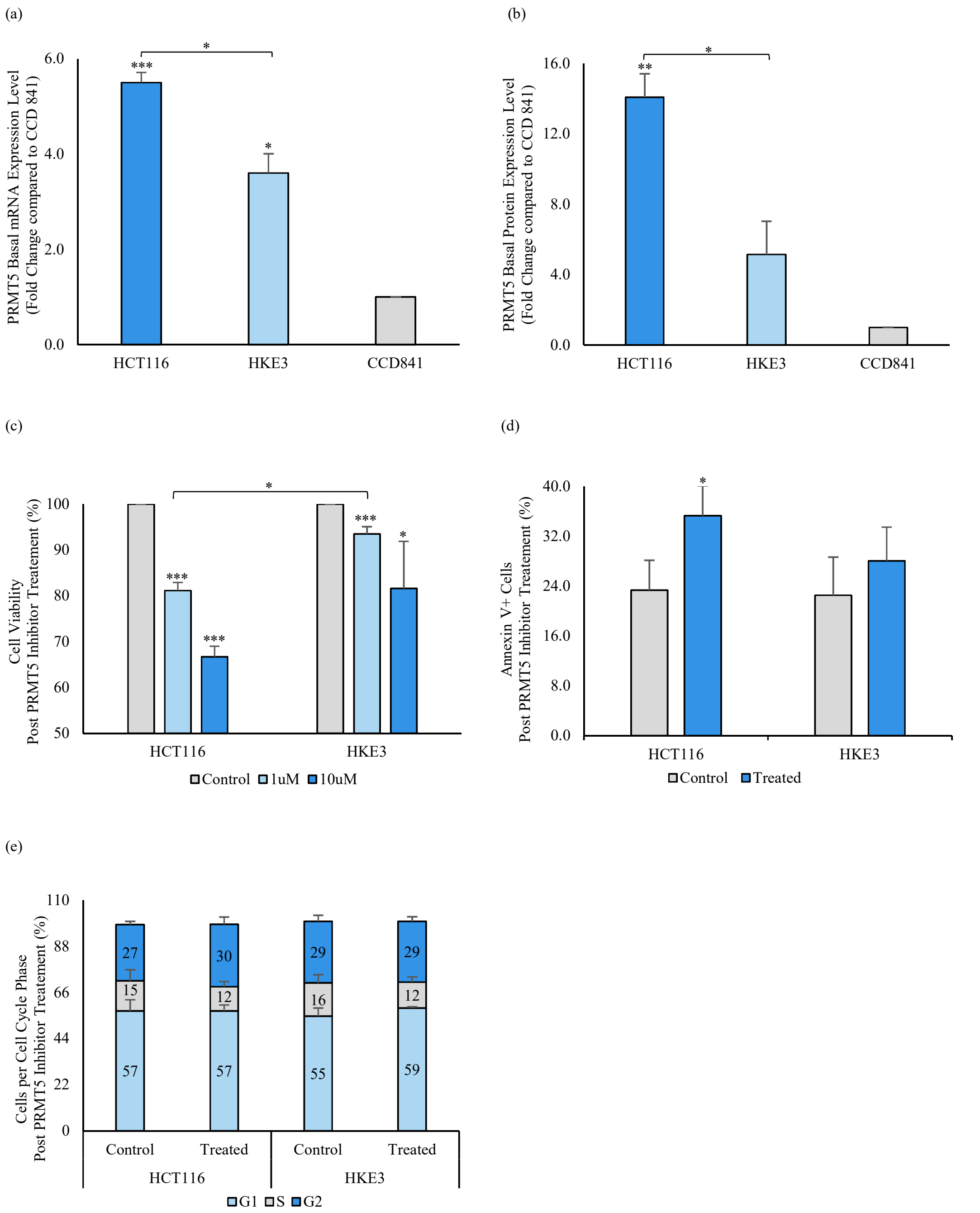


**Figure S2.** Experimental data for isogenic cell lines HCT116 (*KRAS* mutant) and HKE3 (*KRAS* WT). (**a**) Quantified qPCR assay results showing basal PRMT5 expression of HCT116 and HKE3 cell lines at the transcriptional level; (**b**) Quantified western blot assay results displaying the basal PRMT5 expression of HCT116 and HKE3 cell lines at the translational level; (**c**) Quantified cell viability assay results showing the cell viability of HCT116 and HKE3 cell lines post 60 hours of 1μM and 10μM PRMT5 inhibiting treatment; (**d**) Quantified Annexin V assay results displaying the percent of apoptotic cells post 60 h of 10 μM PRMT5 inhibiting treatment in HCT116 and HKE3 cell lines; (**e**) Quantified cell cycle assay results showing the percent of cells per cell cycle phase post 60 h of 10 μM PRMT5 inhibiting treatment in HCT116 and HKE3 cell lines. Data are expressed as means + SD from two to four independent experiments using two CRC cell lines, as well as one normal colon cell line.

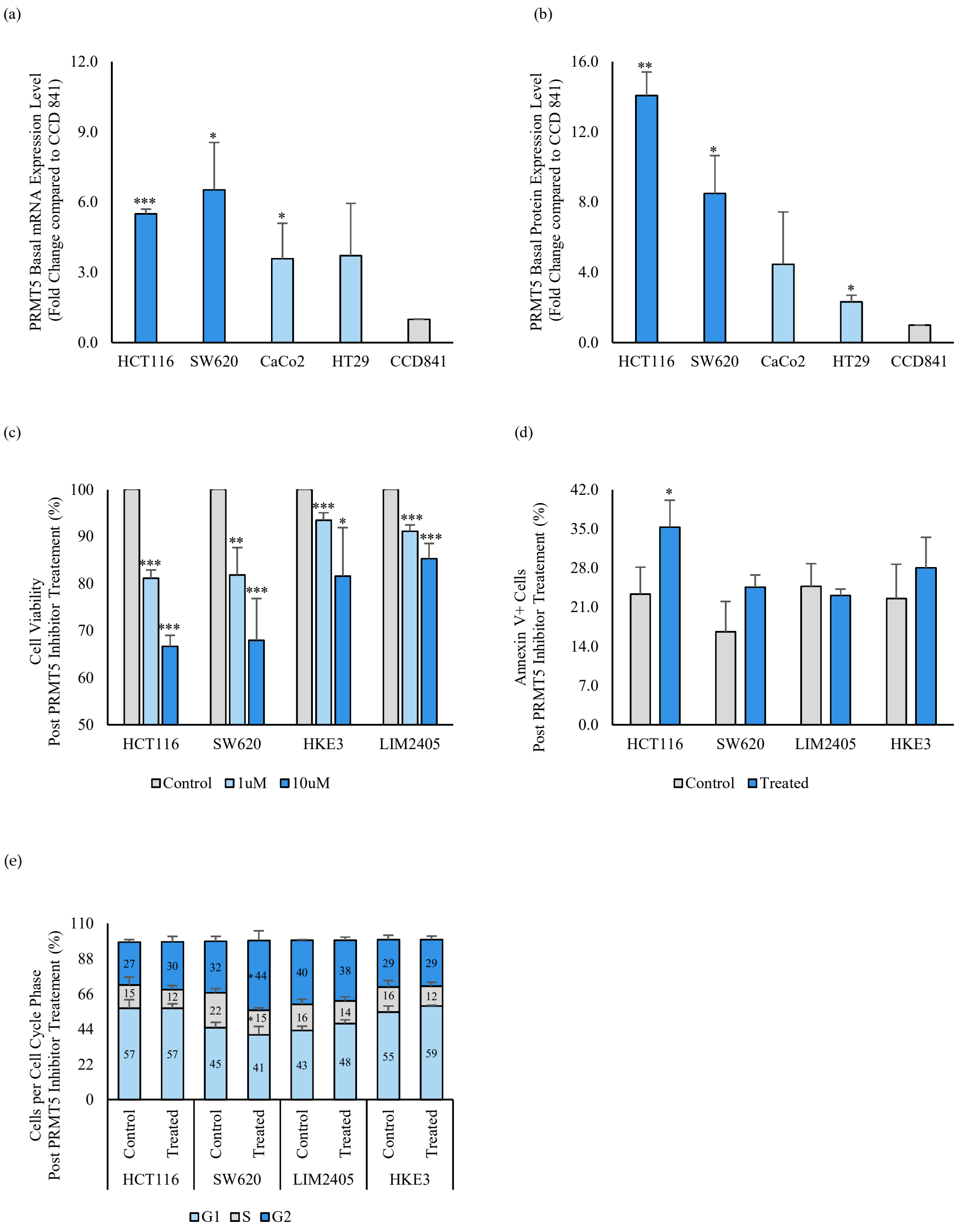


**Figure S3.** Experimental data for the individual cell lines used in Figures 2–6. (**a**) Quantified qPCR assay results showing basal PRMT5 expression at the transcriptional level in CRC cell lines; (**b**) Quantified western blot assay results displaying the basal PRMT5 expression at the translational level in CRC cell lines; (**c**) Quantified cell viability assay results showing the cell viability of CRC cell lines post 60 h of 1 μM and 10 μM PRMT5 inhibiting treatment; (**d**) Quantified Annexin V assay results displaying the percent of apoptotic cells post 60 h of 10 μM PRMT5 inhibiting treatment in CRC cell lines; (**e**) Quantified cell cycle assay results showing the percent of cells per cell cycle phase post 60 hours of 10μM PRMT5 inhibiting treatment in CRC cell lines. Data are expressed as means + SD from two to four independent experiments using four CRC cell lines (2 *KRAS* mutant, 2 *KRAS* WT), as well as one normal colon cell line.

**Table S1.** Percent inhibition of CRC cell lines after treatment with several concentrations of PRMT5 inhibitor.

| PRMT5 Inhibitor Concentration | 1 nM | 10 nM | 100 nM | 1 μM | 10 μM |
| --- | --- | --- | --- | --- | --- |
| LIM2405 Percent Inhibition | −5 | −3 | 0 | 11 | 24 |
| SW620 Percent Inhibition | 1 | 2 | 5 | 14 | 30 |
